# Supplementary material for: Etiologies of Early-Onset Hearing Impairment in Rwanda
Source: Genes (Basel). 2025 Feb 23;16(3):257. doi: 10.3390/genes16030257 (PMC11941765; doi:10.3390/genes16030257)
Supplement: Supplementary file 1 [file genes-16-00257-s001.zip › genes-3476716-supplementary.pdf]

Supplementary materials

Table S1. Primer sequencing for *GJB2* and *GJB6* coding region amplification

Gene	Primer	Primer sequence	Product size	Conditions
GJB2	F4	5′ -GCTTACCCAGACTCAGAGAAG-3′	900	• Annealing temperature: 60°C<br>• Extension temperature: 72°C (25 cycles)
	R1	5′-CTTAATCTAACAACTGGGCAATGC-3′		
GJB6	CDF	5′-TTGGCTTCAGTATGTAATATCACC-3′	990	
	CDR	5′-TCATTTACAAACTCTTCAGGCTACAG-3′		

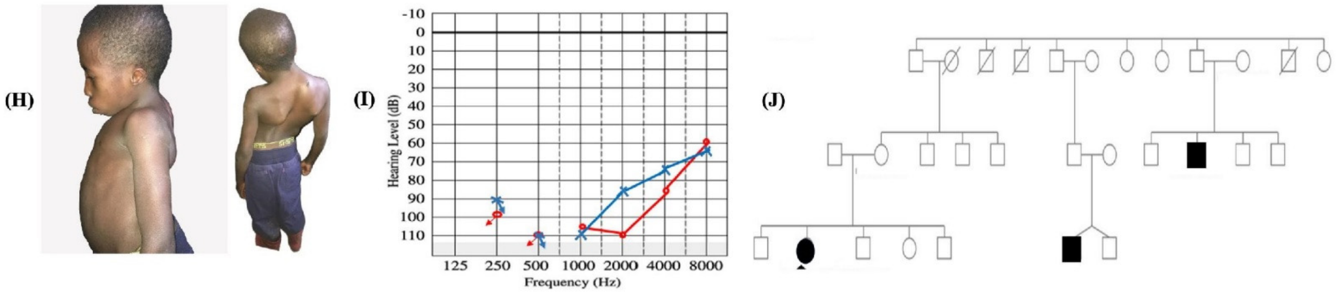

Figure S1. **Clinical profile of a suspected Noonan syndrome case. H**. Familial case with severe HI, left external ear malformation, short neck, and kyphoscoliosis. **I**. Audiogram of the individual in panel **H** showing severe HI. **J**. Pedigree of the presented subject indicates him as the only affected individual in the second generation of the family.

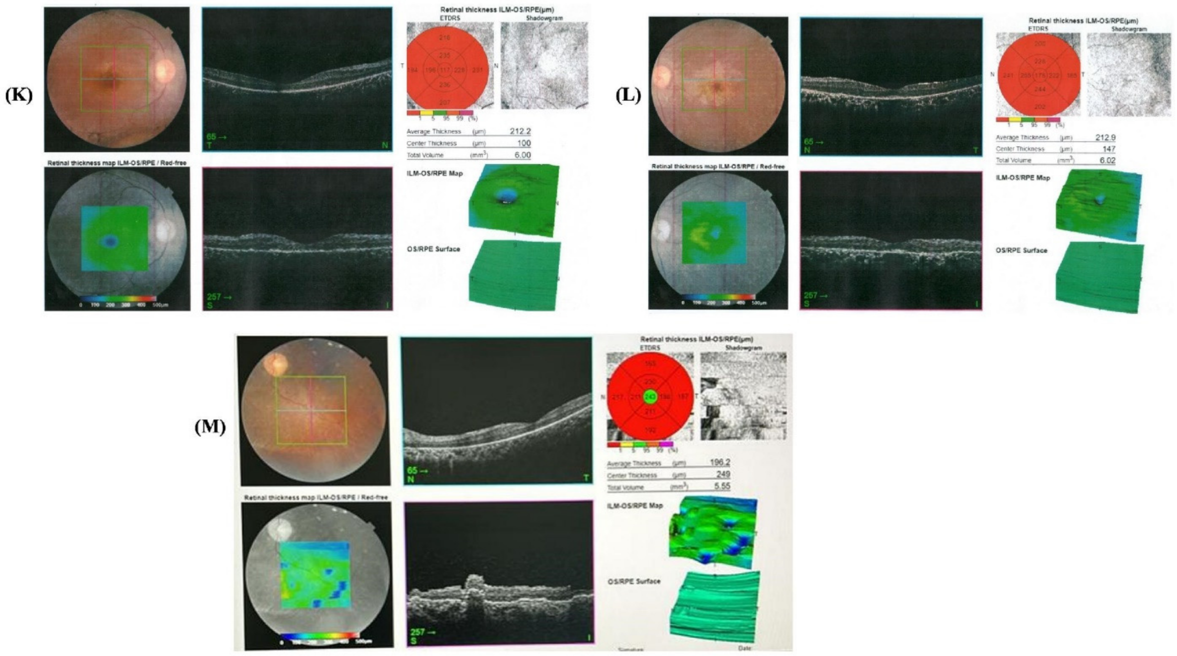

Figure S2. Direct ophthalmoscopy and Ocular Coherence Tomography (OCT) of participants with Usher syndrome. K. and L. Images belong to two sisters showing mild optic disc pallor, scattered peripheral bone-spicule type pigment deposits, and narrowing of retinal arterioles which are typical features of retinitis pigmentosa. There is attenuation of the macular reflex, thinning of the outer retinal layer, and loss of photoreceptors. **M.** Image of a male participant from a different multiplex family, demonstrating retinal pigment epithelial changes and loss of photoreceptors.

Table S2. Clinical data summary of case with putative genetic causes

Characteristic		n	%
**Syndromic or Nonsyndromic**	Syndromic	9	7.37
	Non-Syndromic	113	92.62
**Bilateral vs Unilateral**	Bilateral	82	67.21
	Unilateral	0	0
	Undetermined	40	32.78
**Symmetric vs Asymmetric**	Symmetric	72	59.01
	Asymmetric	10	8.19
	Undetermined	40	32.78
**Degree of HI**	Profound	57	46.72
	Severe	6	4.91
	Moderate-Severe	4	3.27
	Moderate	11	9.01
	Mild	4	3.27
	Undetermined	40	32.78
**Transmission/ Inheritance**	Familial	78	63.93
	Isolated	44	36.06
	Sensorineural	71	58.19
	Conductive	9	7.37
	Mixed	2	1.63
	Undetermined	40	32.78
**Consanguinity**	Yes	2	1.63
	No	120	98.36
	Unknown	2	1.63
